# Supplementary material for: Hippocampal cell- and circuit-specific differences in mitochondrial form and function
Source: bioRxiv. 2025 Dec 17:2025.12.16.694759. Preprint. [Version 1] doi: 10.64898/2025.12.16.694759 (PMC12746136; doi:10.64898/2025.12.16.694759)
Supplement: Supplement 1 [file NIHPP2025.12.16.694759v1-supplement-1.pdf]

bioRxiv preprint doi: https://doi.org/10.64898/2025.12.16.694759; this version posted December 17, 2025. The copyright holder for this preprint (which was not certified by peer review) is the author/funder, who has granted bioRxiv a license to display the preprint in perpetuity. It is made available under aCC-BY 4.0 International license.

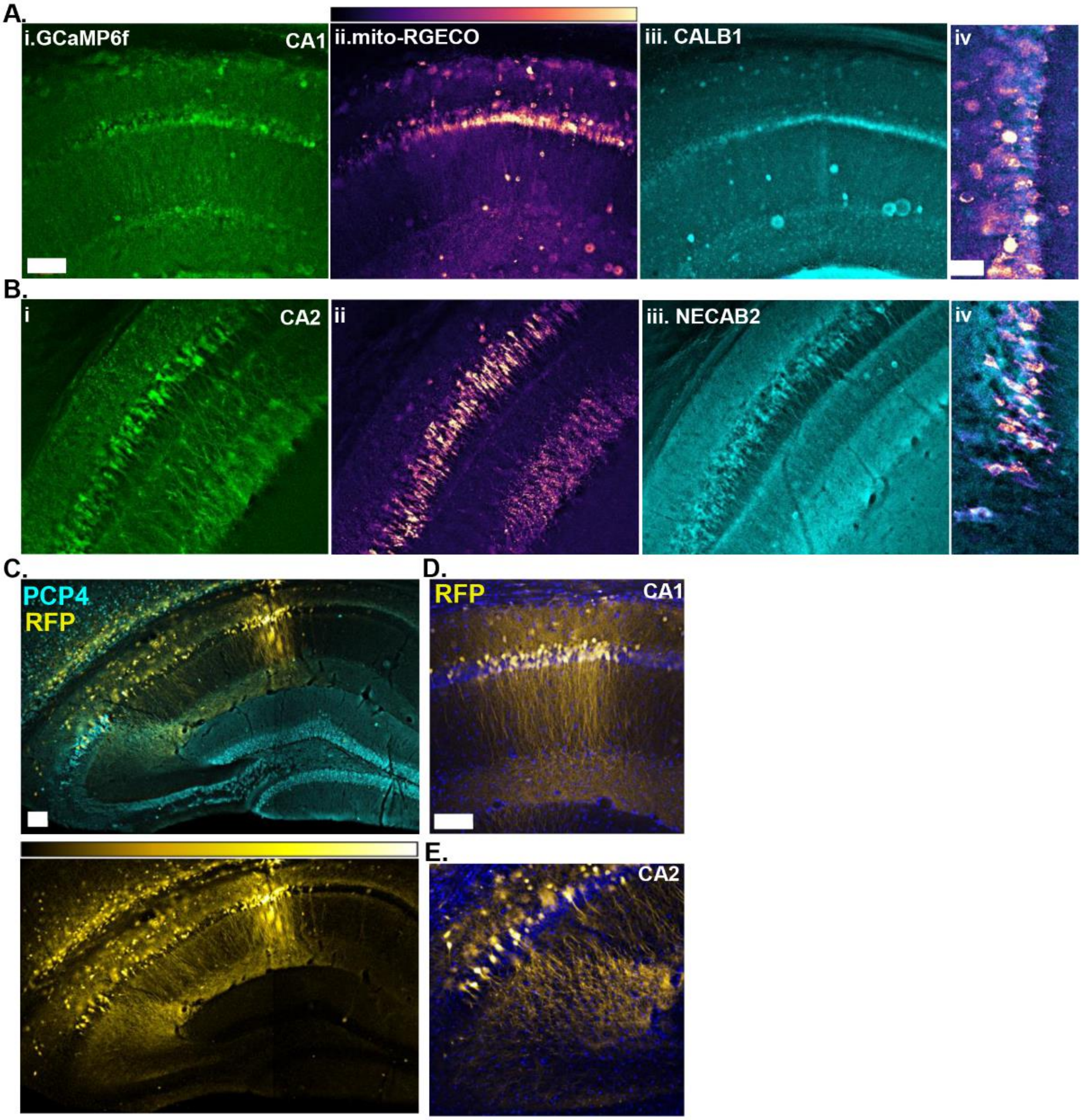

Sup. Fig. 1: Validation of hSyn1-driven CA1 and CA2 mito-RGECO and GCaMP6f labeling.

A. Post-fixed CA1 coronal slice stained for GFP (GCaMP6f, i), RFP (mito-RGECO, ii), and CA1 marker CALB1 (iii). Zoomed inset of SP (iv) to show mito-RGECO expression in CALB1 expressing CA1 neurons.
B. Post-fixed CA2 horizontal slice stained for GFP (GCaMP6f, i), RFP (mito-RGECO, ii), and CA2 marker NECAB2 (iii). Zoomed inset of SP (iv) to show mito-RGECO expression in NECAB2 expressing CA2 neurons.
C. Representative tile image of sparse hSyn1-cre driven tdtomato labeling of CA1 and CA2 neurons in a coronal section. CA2 neurons are labeled with PCP4.
D. Higher magnification of CA1 to show dendritic banding in SLM. Nuclei are labeled with DAPI.
E. Higher magnification of CA2 to show dendrites are out of plane. Nuclei are labeled with DAPI.
Scale bars = (Ai) 100 µm, (Aiv) 50 µm, (C) 100 µm, (D) 100 µm
